# The pilot, proof of concept REMOTE-COVID trial: remote monitoring use in suspected cases of COVID-19 (SARS-CoV 2)

**DOI:** 10.1186/s12889-021-10660-9

**Published:** 2021-04-01

**Authors:** Fahad Mujtaba Iqbal, Meera Joshi, Gary Davies, Sadia Khan, Hutan Ashrafian, Ara Darzi

**Affiliations:** 1 Division of Surgery & Cancer, 10th Floor Queen Elizabeth the Queen Mother Wing (QEQM) St Mary’s Campus, London, W2 1NY UK; 2 West Middlesex University Hospital, Twickenham Road, London, TW7 6AF UK

**Keywords:** Remote sensing technology, Clinical trial, Patient deterioration, Monitoring, ambulatory

## Abstract

**Background:**

SARS-CoV-2 has ever-increasing attributed deaths. Vital sign trends are routinely used to monitor patients with changes in these parameters preceding an adverse event. Wearable sensors can measure vital signs continuously and remotely, outside of hospital facilities, recognising early clinical deterioration. We aim to determine the feasibility & acceptability of remote monitoring systems for quarantined individuals in a hotel suspected of COVID-19.

**Methods:**

A pilot, proof-of-concept, feasibility trial was conducted in engineered hotels near London airports (May–June 2020). Individuals arriving to London with mild suspected COVID-19 symptoms requiring quarantine, as recommended by Public Health England, or healthcare professionals with COVID-19 symptoms unable to isolate at home were eligible. The SensiumVitals™ patch, measuring temperature, heart & respiratory rates, was applied on arrival for the duration of their stay. Alerts were generated when pre-established thresholds were breeched; trained nursing staff could consequently intervene.

**Results:**

Fourteen individuals (M = 7, F = 7) were recruited; the mean age was 34.9 (SD 11) years. Mean length of stay was 3 (SD 1.8) days. In total, 10 vital alerts were generated across 4 participants, resulting in telephone contact, reassurance, or adjustment of the sensor. No individuals required hospitalisation or virtual general practitioner review.

**Discussion:**

This proof-of-concept trial demonstrated the feasibility of a rapidly implemented model of healthcare delivery through remote monitoring during a pandemic at a hotel, acting as an extension to a healthcare trust. Benefits included reduced viral exposure to healthcare staff, with recognition of clinical deterioration through ambulatory, continuous, remote monitoring using a discrete wearable sensor.

**Conclusion:**

Remote monitoring systems can be applied to hotels to deliver healthcare safely in individuals suspected of COVID-19. Further work is required to evaluate this model on a larger scale.

**Trial registration:**

Clinical trials registration information: ClinicalTrials.gov Identifier: NCT04337489 (07/04/2020).

**Supplementary Information:**

The online version contains supplementary material available at 10.1186/s12889-021-10660-9.

## Background

The outbreak of SARS-CoV-2 (COVID-19), declared a pandemic by the World Health Organisation (WHO) and a growing global health problem, has stretched resources, creating pressures within the National Health Service (NHS) with implications for patient safety [1].

In accordance with Public Health England recommendations, a period of isolation may be required for a time duration [2]. The rate of clinical deterioration for individuals suffering from COVID-19 remains unknown; given that widespread vaccine deployment remains imminently unforeseeable, novel strategies are required in approaching this pandemic.

Vital signs trends (heart rate, respiratory rate, blood pressure, temperature, oxygen saturations) are routinely used for monitoring hospital patients [3]. Clinical deterioration may be recognised through changes in these parameters, with prodromal changes preceding an adverse event [4, 5]. Consequently, the National Institute for Health and Care Excellence (NICE) and the Royal College of Physicians (RCP) recommend that all patients have their vital signs recorded every 12 h as a minimum [6, 7].

Across the National Health Service (NHS), the use of the National Early Warning Score 2 (NEWS), a ‘track and trigger’ warning score, has been implemented in accordance with the RCP to guide on escalation protocols and monitoring frequency of vital signs [7]. Accordingly, heart rate (HR), respiratory rate (RR), temperature, blood pressure, oxygen saturation, and level of consciousness are assessed every 4–6 h with more frequent monitoring for acutely unwell patients.

Advances in digital technologies have renewed promise for remote monitoring solutions [8, 9]. Wearable sensors and alerting systems can continuously remotely monitor vital parameters, recognising early deterioration and supporting clinical decision making. As such, allowing individuals to receive care outside of expensive hospital facilities; in resource limited hospitals; and in alternate sites during crises [10].

Delivery of healthcare outside of hospital facilities (e.g. in hotels) is theoretically possible through continuous remote monitoring of vital signs but has yet to be studied; given the global pandemic and fear of future waves, assessment of its viability is justified. As such, the aim of this study was to evaluate the practicality of the SensiumVitals™ system in delivering healthcare in a hotel, acting as an extension to a healthcare trust for individuals suspected of coronavirus. This pilot was performed to determine the feasibility of implementing this system to support future studies, with a focus on wearability, data usability, and safety. This will inform a further definitive trial, optimising recruitment, and follow-up protocols.

## Methods

### Study design

This pragmatically designed, observational, unblinded, pilot and feasibility study was conducted in hotels nearby London airports from May 2020 to June 2020. A detailed protocol has been published [11]. Briefly, returning travellers into London airports or healthcare staff who were suspected of having COVID-19 and were unable to isolate, were recruited into hotels for isolation, and eligible for the study. Exclusion criteria included the presence of a pacemaker, skin reaction to the wearable patch, and consent withdrawal.

The duration of isolation varied, in accordance with changing government guidelines, swab results, and symptomatology. These individuals were assessed by healthcare professionals, swabbed if necessary, and fitted with a wearable patch before being securely transferred to their rooms. A central monitoring hub was established to monitor the recorded parameters by the National Health Service (NHS) healthcare staff from a local Trust. The hub consisted of a site manager, porters, security staff, nurses, ambulance services, professional cleaners, and hotel staff.

All participants provided informed consent and were followed for the duration of their stay. Ethical approval for this study was granted by the Research Ethics Committee (IRAS: 281757). The trial was performed in accordance with Good Clinical Practice guidelines and the Declaration of Helsinki. Patient data was anonymised to ensure privacy. Storage and handling of personal data complied with the General Data Protection Regulation.

### Wearable sensor and alerts

**Fig. 1:**
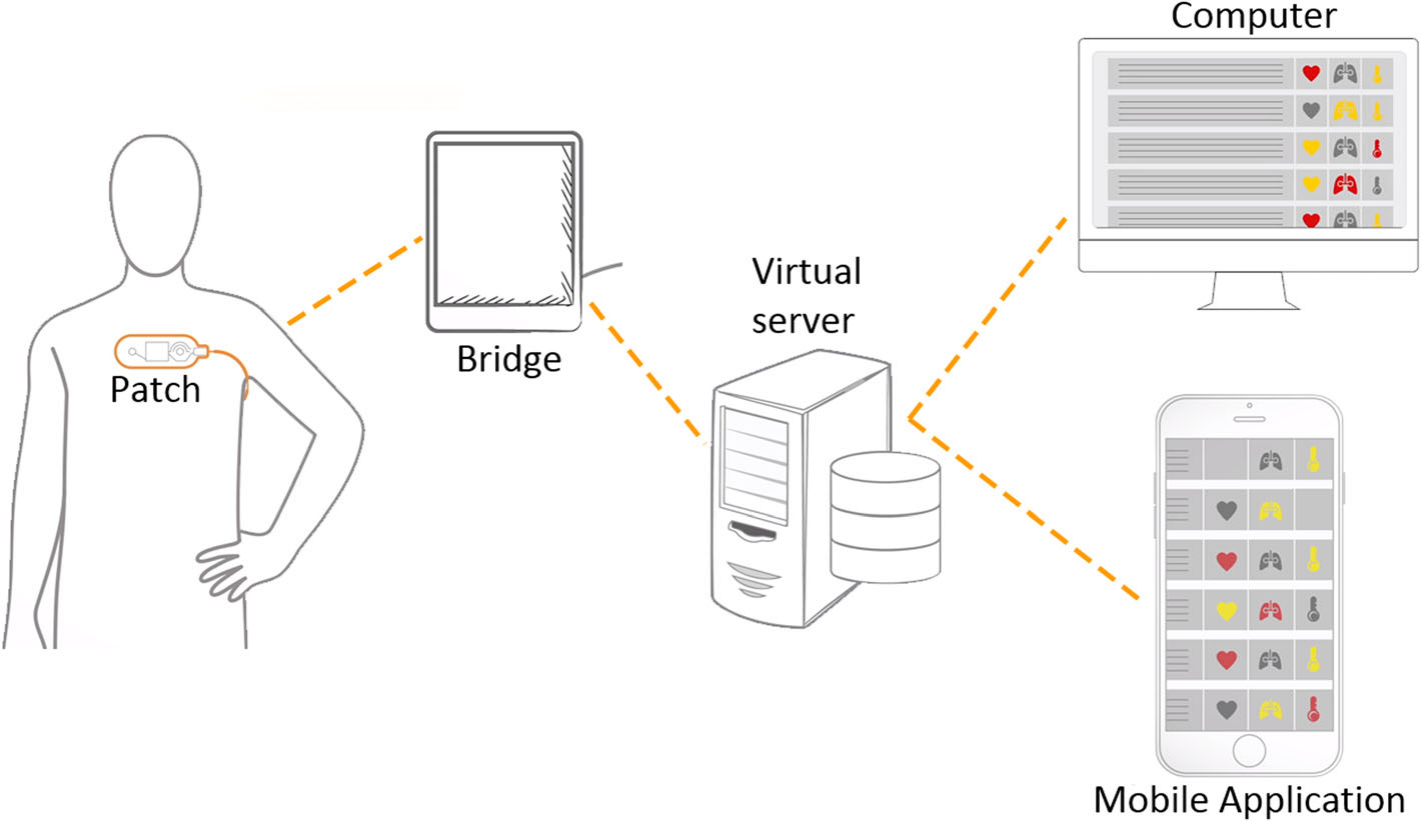
SensiumVitals monitoring system; permission granted to use image by SensiumVitals

SensiumVitals™ have produced a disposable, lightweight, waterproof, wearable wireless ‘patch’ which attaches to a participant’s chest with two adhesive ECG electrodes and records HR, RR and axillary temperature every 2 min, transmitting the data to a central monitoring hub, viewable through a secured web-browser, through radiofrequency and dedicated intranet hotspots (bridges) installed in hotel rooms (Fig. 1). This provided continuous monitoring for individuals at the hotel. It is a low powered device with a battery life of 5 days.

RR was recorded using principles of impedance pneumography and HR through single-lead ECG. A temperature-sensitive resistor is placed in the axilla for readings. Once a physiological signal is acquired, it is processed by embedded algorithms within the sensor to ensure noisy or irregular signals are not reported, reducing false alerts.

All alerts were viewed by dedicated trained nurses at a central monitoring hub, providing continuous, on-site cover at the hotel. Alert transmissions were generated when predefined alarm thresholds for vital parameters were crossed [11].

### Intervention protocol for alerts

All incoming alerts were deemed to be of potential clinical relevance and resulted in a phone contact between the interpreting nurse and the participant; each hotel room had installed landlines. This allowed for additional question seeking at the discretion of the health care worker to gain insight. Potential outcomes included administration of analgesia, general education and anxiety management, virtual GP review, or escalation to the hospital. These actions were protocol driven.

### Outcome measures

The total number of alerts, proportion of actioned alerts, and resultant actions (i.e. phone consultation, virtual general practitioner review, transfer to hospital) were measured.

To understand the acceptability and usability of the SensiumVitals™ system by participants and healthcare staff, mixed methods consisting of semi-structured interviews and questionnaires were utilised. For participants, these were completed at the end of their isolation; for healthcare staff, they were conducted once familiarity with the system was established. The questionnaires consisted of five-point Likert scale responses (strongly disagree to strongly agree), with elements adapted from the validated System Usability Scale (supplementary material) [12]. Semi-structured interviews, conducted using prepared topic guides, were recorded, anonymised, and transcribed verbatim before entered in to NVivo 12 for analysis (supplementary material). Topics covered included comfort, understanding, safety, and repeated use.

### Statistical analysis

Descriptive statistics were used to describe baseline characteristics of participants, alerting frequencies, and events.

A mixed methods analysis was undertaken for questionnaire and semi-structured interview data. Frequency distributions were generated for Likert scale responses. Interview transcripts were analysed using Braun and Clarke’s thematic analysis [13]. In brief, the transcripts were independently studied by two researchers, evaluating for common attitudes and experiences between participants. Emergent themes were coded with data systematically reviewed to ensure the identified themes were suitable. Facilitators and barriers were ascertained from healthcare staff [14, 15].

## Results

### Study population

**Fig. 2:**
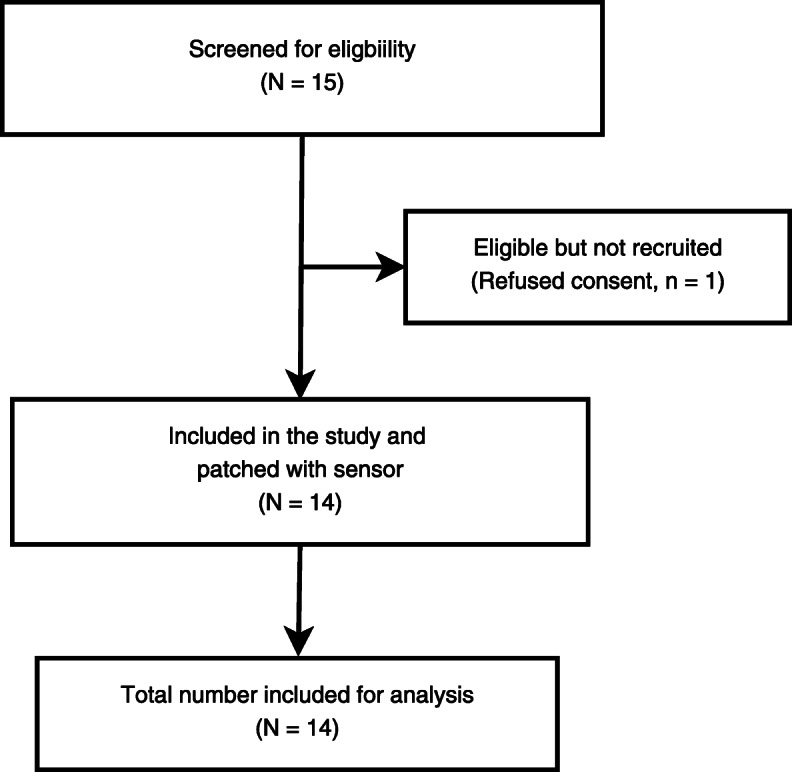
Participant flow diagram

A total of 15 participants were eligible for the study: of which, one refused study participation stating anxiety as a reason. The remaining were enrolled into the study (Fig. 2, *N* = 14). Baseline demographics are presented in Table 1. The mean length of stay at the hotel was 3.1 (SD: 1.8) days.

**Table 1:** Baseline characteristics

Characteristics (N = 14)	N (%)
Age (years), mean (SD)	34.9 (11.0)
Male	7 (50)
Female	7 (50)
Caucasian	7 (50)
BAME	7 (50)
SARS-CoV 2 swab positive	3 (21.4)
Co-morbidity: • None	6 (42.9)
• Asthma	3 (21.4)
• Cardiovascular (e.g. hypertension)	2 (14.3)
• Neurological (e.g. migraine, seizures)	2 (14.3)
• Anxiety/depression	2 (14.3)

*BAME* British, Asian, Minority Ethnic

### Clinical events

During the study, a total of 10 vital alerts were received across four individuals (Table 2). Two alerts were unactioned (for abnormal respiratory rates) and expired. None of the recruited individuals required hospitalisation or virtual general practitioner review. Two individuals developed a skin reaction to the adhesive (tape or ECG electrodes) but continued to participate in the study. There were no dropouts in our study.

**Table 2:** Clinical events following alerts

Events	Alert	Management
10 vital alerts in 4 patients	Abnormal temperature reading (1 episode)	Anti-pyretic (paracetamol) administered following telephone consultation (1 episode)
	Abnormal respiratory rate reading (9 episodes)	No action taken following telephone review (6 episodes) Electrodes reapplied (1 episode)

### Healthcare and participant perceptions

#### Participant perceptions

**Fig. 3:**
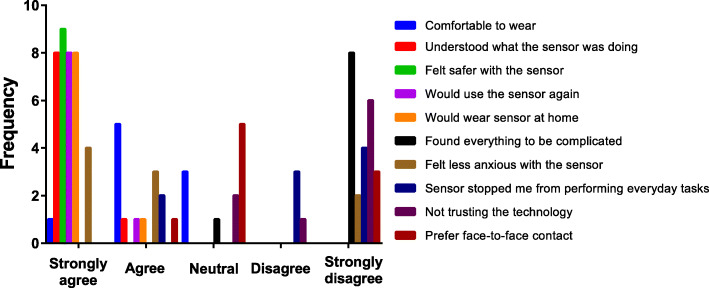
frequency distribution of participant questionnaire responses

Nine participants responded to questionnaires and four participated in semi-structured interviews. Overall, guests perceived the sensor to be comfortable, felt safer with its use, and would wear the sensor again; very few found it to be complicated. Frequency distribution of participant responses are shown in Fig. 3.

Four main themes emerged from the interviews: i) functionality; ii) comfort and usability; iii) sense of security; and iv) privacy.

#### Functionality

Overall, participants were aware of the purpose of monitoring and the miniturisation of the sensor was appealing.

“ … monitor you remotely from the [central monitoring] station … something that’s an alteration or a shift [in your vital signs], [the nurses] can then contact us and see how we’re feeling and if it matches [the vital sign readings] … you can’t really see [the sensor through] the clothes. It’s quite small … you can’t see it” (guest 1)

“it was not heavy at all.” (guest 2)

One obvious advantage was the reduction in viral exposure to healthcare staff and this was reflected by participants.

“you can have limited contact to patients, especially if the patient is infectious. I’d say that you can monitor the patient [without] having frequent contact … so it’s good.” (guest 2)

#### Comfort and usability

Most guests reported the sensor as comfortable to wear in the questionnaire. However, an initial period may be required to be accustomed to the sensor.

“it’s not really that uncomfortable … I got used to it over time. It’s just about adjusting to it the first time I wore it.” (guest 2)

Guests reported mixed experiences on the practicalities of wearing the sensor with their daily activities.

“I tended not to sleep on my side as I thought it might come off. It wasn’t particularly restrictive [otherwise]. (guest 3)

“when I shower I’m always very cautious to wash around it.” (guest 1)

“I think the tape [to fasten the temperature wire] was a little bit [inconvenient] but not the actual wire” (guest 3)

“At first it was difficult to adjust [to]. It was like it felt a bit tight, I mean the tape on my underarm was a bit tight … also I did wonder the probe to lose contact with my skin, because I wanted the monitoring to be precise. And so I was always worried about losing contact with the probe on my skin … over time it was comfortable … I got used to it over time … washing or sleeping wasn’t a problem” (guest 4)

“I took it off [before] I had my shower … I would have probably been a bit reluctant to shower in it.” (guest 3)

#### Sense of security

Participants felt comforted and secure knowing they were receiving continuous monitoring remotely.

“I felt, to be honest, probably a bit more comfortable that somebody’s keeping an eye on things … things can deteriorate quickly.“ (guest 3)

“I feel secure and I felt safe being there, being monitored” (guest 2)

“It felt good. It gave me a little bit more of a sense of being monitored and cared for.” (guest 4)

#### Privacy

None of the guests felt their privacy was invaded through sensor use.

“it’s not too intrusive of what my activities are” (guest 1)

#### Healthcare staff perceptions

A total of six staff members responded to the questionnaire and participated in semi-structured interviews.

**Fig. 4:**
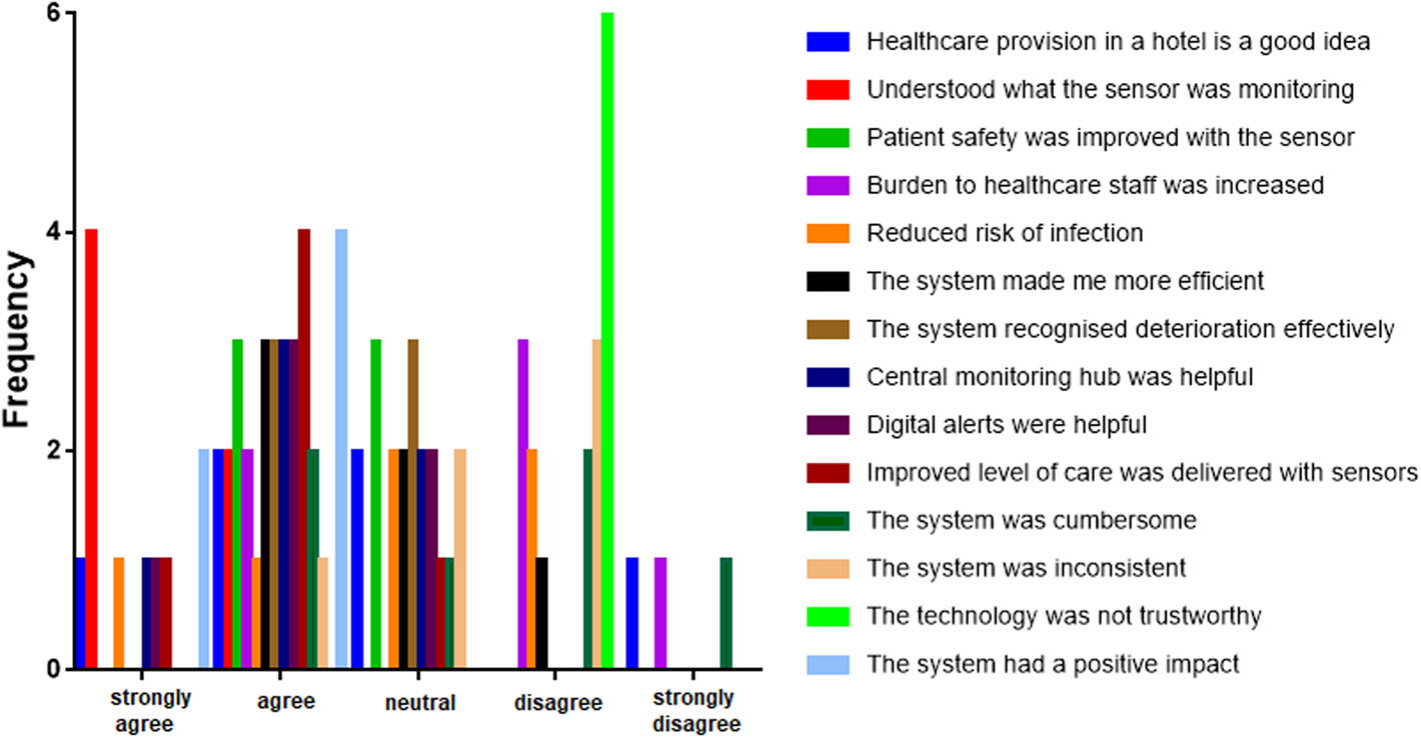
frequency distribution of healthcare questionnaire responses

The hotel model of healthcare provision was perceived with mixed feedback amongst nursing staff. However, most staff felt that the technology was trustworthy, not burdensome, and delivered an improved level of care because of wearable sensors and digital alerting. Frequency distribution responses are shown in Fig. 4.

Two main themes emerged from the interviews which were sub-categorised into facilitators and barriers: i) factors relating to the sensor and ii) perceived usefulness.

#### Factors relating to the sensor

##### Facilitators

Overall, healthcare nurses favoured the dimensions of the sensor and the simplicity of the system.

“It was quite compact, quite small. Easy to apply” (nurse 6)

“portability … the individual patients/guests are not restricted in their movements … [the system] was quite intuitive and quite easy” (nurse 2)

“person doesn’t seem to know they’re wearing it, so that’s good” (nurse 3)

“I’m quite surprised actually that they could carry on with the routine activities, even shower with it, without having to remove it and reapply it which I thought was fantastic … . I think the guests liked it too. I know one guy who came, a young gentleman, he was really glad that we were monitoring him” (nurse 6)

##### Barriers

Adhesive tapes and electrodes were noted to have varying efficacy amongst different guests, affecting overall signal quality. This required repeated fastening and replacement in some cases.

“we had one person who the electrodes kept popping off so that might be something [that needs] improving.” (nurse 2)

“I think a weakness was the temperature probe.” (nurse 4)

“There are one or two guests that the readings were not coming out as we would have liked. And it could be because they were moving very fast or they have removed [the sensor] from its position.” (nurse 5)

#### Perceived usefulness

##### Facilitators

The system was perceived as providing a clinical insight by healthcare staff whilst reducing viral exposure.

“I think for our group of patients it’s been really good. Because they’re behind a closed door it has given us an opening to see what else is going on” (nurse 3)

“It’s quite beneficial. It saves time and if the machine is accurate and that it will trigger any intervention if necessary and I think it’s a good thing.” (nurse 5)

“gave me additional reassurance that we knew exactly what was going on” (nurse 6)

“easier to sort of escalate if people needed further treatment quickly” (nurse 1)

“I think staff exposure [to coronavirus] was reduced” (nurse 6)

“it gives them [nurses] time to be able to do other things with them.” (nurse 3)

##### Barriers

Nurses reported the need for trained staff to action alerts and, for some individuals, may be a source of anxiety.

“that is a safety issue, people need to know how to use things properly, how to escalate the information it’s providing them with properly because to not use it puts the patient at risk.” (nurse 2)

“You still need sort of trained staff to understand what it all means so you wouldn’t be able to run it here with a healthcare assistant or with no nurses, you’d need to have a healthcare professional that was experienced here.” (nurse 1)

“there may be some patients where mental health may be the dominant thing that you’re trying to treat … wearing this patch may feed into a paranoia or a confusion or something like that.” (nurse 2)

## Discussion

This trial demonstrated feasibility and proof of concept of a rapidly implemented model of healthcare delivery through remote monitoring during the first wave of COVID-19 whereby hotels acted as an extension to a healthcare trust. Digital alerts generated when pre-established thresholds were breeched allowed for healthcare professionals to interpret and respond successfully. This study also provided a broad overview of participant and healthcare staff perceptions on digital alerting and sensor use in a remote monitoring context. Although previous work has explored patient attitudes of sensor use in hospital settings, healthcare staff perceptions were not described and remote monitoring significantly alters the delivery of healthcare, a model which undergoes further evolution during a pandemic [16, 17].

Although a proportion of generated alerts resulted in clinical action, no individuals in our cohort required virtual GP review or hospitalisation. Given our small sample size and limited duration of stay at the hotel, the potential benefits of digital alerting systems and continuous remote monitoring may have been underestimated. However, our cohort was relatively young with few co-morbidities, representing a low potential to require escalation for hospital care. Nonetheless, given the exploratory nature of the trial, feasibility was demonstrated with the clinical events observed and no dropouts.

Previous work has shown acceptability and practicability of continuous monitoring using wearable sensors on general surgical and medical wards [18, 19]. Initial work by Downey et al. which also tested the same sensor (SensiumVitals™) were limited by imbalanced randomisation across the two trial arms [18]. Nonetheless, the trials demonstrated feasibility in hospital settings. Barriers and facilitators of implementing wearable sensor based continuous monitoring were conducted on a surgical cohort which similarly identified the importance of design, comfort, and safety; semi-structured interviews for patients and healthcare staff, favoured the notion of continuous vital sign monitoring in general wards [19]. It should be noted that many of the patients interviewed were admitted for malignant disease which is likely to influence qualitative perceptions. Moreover, practicality of these systems utilised in hospital care is vastly different to a remote & community healthcare model. Our work identified additional perceptions in remote sensing environments.

Pre-pandemic, prominent global health leaders and societies have focused on delivering ‘high-value care’. The American College of Physicians (ACP), the Society of Hospital Medicine (SHM), Royal College of Physicians (RCP), Royal College of General Practitioners (RCGP), and leaders in the field of medicine advocated the use of high-value care [20–22]. This dictates a judicious use of resources while providing the best possible care. With the scarcity of resources in the current pandemic, our study can envisage potential future implications.

Despite the strengths of our study, the design presents inherent limitations. To maximise capacity at the hotel given the unknown of the pandemic, a pragmatic, observational design was favoured; randomisation was not deemed appropriate. Government restrictions rapidly underwent changes for air travel and isolation guidelines, which significantly altered our sample size and duration of isolation. Moreover, the inclusion of healthcare staff who wore the sensor, are inherently familiar with vital signs and clinical observations; this may bias the description of favourable experiences. However, such individuals are also likely to carry greater expectations. As such, their inclusion is unlikely to affect the demonstration of proof of principle. Device specific outcomes, such as the varying efficacy of adhesive tapes and electrodes, lack generalisability when compared to other available sensors but provide a broad insight into design considerations.

In conclusion, feasibility of remote sensing was demonstrated in our trial and favourable experiences described by healthcare staff and participants. Wearable sensors providing continuous monitoring may facilitate predictive modelling for deterioration and early interventions in remote & community settings. Further work should explore the effect of remote sensing and its impact on clinical outcomes, particularly given the evolving model of healthcare delivery, accelerated by the pandemic.

## Supplementary Information

**Additional file 1.**

## Data Availability

The datasets generated during and/or analysed during the current study are available from the corresponding author on reasonable request.
