# Supplementary material for: The pilot, proof of concept REMOTE-COVID trial: remote monitoring use in suspected cases of COVID-19 (SARS-CoV 2)
Source: BMC Public Health. 2021 Apr 1;21:638. doi: 10.1186/s12889-021-10660-9 (PMC8013165; doi:10.1186/s12889-021-10660-9)
Supplement: Supplementary file 1 — Additional file 1. [file 12889_2021_10660_MOESM1_ESM.docx]

REmote MOniToring usE in suspected cases of COVID-19 (SARS-CoV 2): Pilot, proof of concept? The REMOTE-COVID trial

*Fahad Mujtaba Iqbal^1*^, Meera Joshi^1^, Gary Davies^2^, Sadia Khan^2^, Hutan Ashrafian^1^, Ara Darzi^1^*

1. Division of Surgery & Cancer, 10^th^ Floor Queen Elizabeth the Queen Mother Wing (QEQM) St Mary’s Campus, London, W2 1NY
2. West Middlesex University Hospital, Twickenham Road, Isleworth, TW7 6AF

# Supplementary material

## Questionnaire for participants

| **Statement** | **Strongly agree** | **Agree** | **Neutral** | **Disagree** | **Strongly Disagree** |
| --- | --- | --- | --- | --- | --- |
| I found the sensor comfortable to wear |  |  |  |  |  |
| I understood what the sensor was doing |  |  |  |  |  |
| I felt safer with the sensor on |  |  |  |  |  |
| I would use the sensor again |  |  |  |  |  |
| I would wear the sensor at home |  |  |  |  |  |
| I found everything to be complicated |  |  |  |  |  |
| I felt less anxious with the sensor on |  |  |  |  |  |
| I felt the sensor stopped me from performing my normal everyday tasks |  |  |  |  |  |
| I did not trust the technology in doing its job |  |  |  |  |  |
| I prefer face-to-face contact rather than relying on technology |  |  |  |  |  |

## Questionnaire for healthcare staff

| Healthcare provision in a hotel is a good idea |  |  |  |  |  |
| --- | --- | --- | --- | --- | --- |
| I understood what the sensor was monitoring |  |  |  |  |  |
| Patient safety was improved with this technology |  |  |  |  |  |
| The sensor increased burden to healthcare staff |  |  |  |  |  |
| I felt less at risk of infection because of the sensor |  |  |  |  |  |
| This system made me more efficient (e.g. reducing personal protective equipment use) |  |  |  |  |  |
| This technology was good at recognising when someone deteriorates |  |  |  |  |  |
| The central monitoring hub was helpful |  |  |  |  |  |
| The digital alerting/notifications were helpful |  |  |  |  |  |
| The level of care provided to individuals was better with the use of sensors |  |  |  |  |  |
| I found the system cumbersome |  |  |  |  |  |
| There was too much inconsistency with the system |  |  |  |  |  |
| I did not trust the technology in doing its job |  |  |  |  |  |
| Overall, I felt this system had a positive impact |  |  |  |  |  |
